# China Sets Up its First Doctors’ Day: To Honor the Contributions Made by Chinese Doctors in the Public Health Work

**DOI:** 10.18502/ijph.v49i7.3597

**Published:** 2020-07

**Authors:** Ce ZHOU, Fu-Rong ZHANG, Yan-Li LIAO, Jian KANG

**Affiliations:** 1. Department of Proctology, Hospital of Chengdu University of Traditional Chinese Medicine, Chengdu, China; 2. School of Acupuncture and Tuina, Chengdu University of Traditional Chinese Medicine, Chengdu, China; 3. Department of Obstetrics, Sichuan Jinxin Women and Children's Hospital, Chengdu, China

## Dear Editor-in-Chief

In Aug 19, 2018, China celebrated its first national Doctors’ Day, approved and supported by the State Council. The main purpose of setting up the festival is to honor the clinical physicians and to pay tribute to their indispensable contributions in Chinese public health work. This action will encourage our society to pay more respects and attention to physicians and the healthcare sector, which will inspire all the medical staff in this country.

The day means much more than just a common festival for Chinese doctors. It is an official recognition and appreciation of their hard and even sacrificing work at the state level. During the past decade, China’s medical reform has confronted with many though challenges. China now owns a population of more than 1.39 billion people, however, the total number of Chinese registered medical practitioners is only 3.39 million, which brings about a huge gap between medical demand and supply in China. To solve the severe shortage of medical personnel, the Chinese government has made great efforts by taking following measures, such as to increase budget on medical development, improve the income of medical staff, enlarge the enrollment of medical students and implement some other preferential policies. The huge gap between the medical need and supply has also aggravated their working load, putting much extra pressure on Chinese medical workers, and further results in the shortage. According to the latest two studies, the workload of Chinese physicians has been on the increase (1). There seems to be a rising trend of the sudden death of the Chinese physician as well (2). Over the years, the Chinese medical evaluation and promotion system have overemphasized on doctors’ scientific achievements, including acquiring research projects and publishing academic papers. Both the heavy clinical and academic workload has made them undergo over stress physically and mentally. To make it worse, misleading information from the mass media has abetted medical disputes even physical conflicts between patients and doctors in the hospital (3). Along with some sensational negative news in this field, such as the 2013 GlaxoSmithKline bribery incident and 2018 Chinese vaccine scandals (4), have drastically blew way the last straw of their mutual trust. The already tensed doctor-patient relationship has upgraded and become a big social problem in China (5). Under the circumstance, some physicians and nurses have even decided to change their jobs, which in turn enlarge the gap. Without proper settlement, the gap will exert tremendous influence on both Chinese and international society. According to the latest Chinese Statistical Yearbook (http://www.stats.gov.cn/tjsj/ndsj/2017/indexch.htm), China owns nearly one-fifth of world’s population, ranking first worldwide and aging population proportion is estimated to 23.3% in 2050, which will cause great burdens on national and global health care and social economy. China's public health work will continue to face enormous challenges in the future, and among all the causes, the absolute shortage of physicians comes first. The establishment of the Doctors’ Day means that the Chinese government values more on the indispensable role of physicians in public health work. High-quality public health construction is a complex and systematic project, setting up the Doctors’ Day is a good beginning which shows the determination of the Chinese government in the cause. This initiative may be useful for some other countries, especially for those developing countries lacking experience in public health construction.
